# Checklist of the family Culicidae (Diptera) in Finland

**DOI:** 10.3897/zookeys.441.7743

**Published:** 2014-09-19

**Authors:** Larry Huldén, Lena Huldén

**Affiliations:** 1 Finnish Museum of Natural History, Zoology Unit, P.O. Box 17, FI-00014 University of Helsinki, Finland; 2 University of Helsinki, Department of Agricultural Sciences, P.O. Box 27, FI-00014 University of Helsinki, Finland

**Keywords:** Checklist, Finland, Diptera, Culicidae, mosquitoes

## Abstract

A checklist of the Culicidae (Diptera) recorded from Finland is provided.

## Introduction

The family Culicidae comprises 3539 extant species world-wide. The Finnish fauna is relatively small in relation to world fauna. Because of medical importance this family has been extensively taxonomically studied but still new species are discovered. The generic and subgeneric classifications of Culicidae follow Reinert et al. 2009. Because of some confusing use of names in the first half of the twentieth century in the Finnish literature, all available names are included in the list for clarity. The most important work on the Finnish mosquitoes was done by Pirkka Utrio (1975, 1976, 1977, 1978, 1979).

**Number of species:**

World: 3539 extant species + 25 fossil species (Harbach 2014)

Europe: 104 species (Snow and Ramsdale 2004)

Finland: 38 species

## Checklist

suborder Nematocera Dumeril, 1805

infraorder Culicomorpha Hennig, 1948

superfamily Culicoidea Meigen, 1818

**CULICIDAE** Meigen, 1818

ANOPHELINAE Grassi, 1900

***Anopheles*** Meigen, 1818

**sg. *Anopheles*** Meigen, 1818

*Anopheles beklemishevi* Stegni & Kabanova, 1976

= *maculipennis* auct. in part

*Anopheles claviger* (Meigen, 1804)

*Anopheles messeae* Falleroni, 1926

= *claviger* (of Fabricius, 1805) auct. nec Meigen, 1804

= *maculipennis* auct. in part

CULICINAE Meigen, 1818

***Aedes*** Meigen, 1818

*Aedes cinereus* Meigen, 1818

***Aedimorphus*** Theobald, 1903

*Aedes vexans* (Meigen, 1830)

***Coquillettidia*** Dyar, 1905

*Coquillettidia richiardii* (Ficalbi, 1889)

***Culex*** Linnaeus, 1758

**sg. *Culex*** Linnaeus, 1758

*Culex pipiens* Linnaeus, 1758

*Culex torrentium* Martini, 1925

**sg. *Neoculex*** Dyar, 1905

*Culex territans* Walker, 1856

= *apicalis* auct. nec Adams, 1903

***Culiseta*** Felt 1904

= *Theobaldia* Neveu-Lemaire, 1902 preocc.

**sg. *Culicella*** Felt, 1904

*Culiseta morsitans* (Theobald, 1901)

*Culiseta ochroptera* Peus, 1935

**sg. *Culiseta*** Felt, 1904

*Culiseta alaskaensis* (Ludlow, 1906)

*Culiseta annulata* (Schrank, 1776)

*Culiseta bergrothi* (Edwards, 1921)

= *glaphyroptera* auct. nec (Shiner, 1864)

*Culiseta subochrea* (Edwards, 1921)

***Dahliana*** Reinert, Harbach & Kitching, 2006

*Dahliana geniculata* (Olivier, 1791)

***Ochlerotatus*** Lynch Arribalzaga, 1881

**sg. *Ochlerotatus*** Lynch Arribalzaga, 1881

*Ochlerotatus annulipes* (Meigen, 1830)

*Ochlerotatus euedes* (Howard, Dyar & Knab, 1913)

= *beklemishevi* Denisova, 1955

*Ochlerotatus cantans* (Meigen, 1818)

= *waterhousei* (Theobald in Waterhouse, 1905)

= *maculatus* auct. nec (Meigen, 1804)

= *rusticus* auct. nec (Rossi,1790)

*Ochlerotatus caspius* (Pallas, 1771)

*Ochlerotatus cataphylla* Dyar, 1916

*Ochlerotatus communis* (De Geer, 1776)

= *nemorosus* (Meigen, 1818)

*Ochlerotatus cyprius* Ludlow, 1920

= *freyi* Edwards, 1921

*Ochlerotatus diantaeus* Howard, Dyar & Knab, 1912

*Ochlerotatus dorsalis* (Meigen, 1830)

*Ochlerotatus excrucians* (Walker, 1848)

*Ochlerotatus flavescens* (Müller, 1776)

= *lutescens* (Fabricius, 1775)

= *variegatus* (Schrank, 1781)

*Ochlerotatus hexodontus* Dyar, 1919

*Ochlerotatus impiger* (Walker, 1848)

= *parvulus* (Edwards 1921)

= *nearcticus* auct. nec (Dyar, 1922)

*Ochlerotatus intrudens* Dyar, 1919

*Ochlerotatus leucomelas* (Meigen, 1804)

*Ochlerotatus nigrinus* (Eckstein, 1918)

= *sticticus* auct. nec Meigen 1828

*Ochlerotatus nigripes* (Zetterstedt, 1838)

= *alpinus* auct. nec (Linnaeus) nomen dubium

*Ochlerotatus pionips* Dyar, 1919

*Ochlerotatus pullatus* (Coquillett, 1904)

*Ochlerotatus punctodes* Dyar, 1922

*Ochlerotatus punctor* (Kirby, 1837)

*Ochlerotatus riparius* Dyar & Knab, 1907

## Notes

***Anopheles maculipennis* Meigen, 1818**. Not recorded in Finland. All previous reports refer to *Anopheles messeae* or *Anopheles beklemishevi*.

***Anopheles claviger*** (of Fabricius, 1805). Mistaken name usage for *Anopheles maculipennis* s. lat. in the early 20^th^ century Finnish literature which then followed the Italian tradition.

**Culex pipiens f. molestus Forskåhl, 1775**. This is not a valid species level taxon. It is a man biting form of the mainly bird biting *Culex pipiens*. Both have been reported from Finland.

***Dahliana geniculata* (Oliver, 1791)**. A possibly occasional specimen of this species was collected in Western Finland, verified by Christine Dahl (Itämies 1981).

***Ochlerotatus alpinus* L.**Frey (1921) included this invalid species name (see Harbach 2014) in the Finnish list with a question mark. Hellén (1931) withdrew it from the Finnish list. According to Utrio (1977), Frey refers to *nigripes* (Zetterstedt, 1838).

***Ochlerotatus behningi* Martini, 1926**. The species is listed for both Finland and Sweden in Dahl (1997). Lundström et al. (2013) do not accept the report for Sweden because of absence of documented locality. Based on the same argument, we do not include this species in the Finnish list.

**Ochlerotatus (Rusticoidus) rusticus (Rossi, 1790)**. Dahl (1997) listed this species for Finland. There are, however, no confirmed collections of the species in Finland. Frey (1921) listed *Aedes maculatus* Meigen (with *Aedes waterhousei* Theobald as a presumed synonym) from Finland. According to Natvig (1948), Meigen made a mistake in 1830 when he stated that “*maculatus* is the male of *cantans*” instead of “*reptans* is the male of *cantans*”, leading to a switch in synonyms. According to Minar (1990) the correct combinations should be as follows: *maculatus* Meigen is a synonym of *rusticus* (Rossi) and both *reptans* (Meigen) and *waterhousei* (Theobald) are synonyms of *cantans* (Meigen). From this context, it is obvious that Frey refers to the common species *cantans* and not to the southern species *rusticus*.

***Ochlerotatus sticticus* (Meigen, 1838)**. The species was reported from Finland (Kilpisjärvi) by Frey (1932) and listed for Finland in Frey and Storå (1941). According to Natvig (1948) and Utrio (1979) this record refers to *Aedes nigrinus* (Eckstein). In addition, it was reported by Syrjämäki (1960) in Finland (Hanko: Tvärminne) but later withdrawn by the author according to Utrio (1979, personal communication). It was also included for Finland in Dahl (1997), probably based on the former reports. According to Gutsevich et al. (1970) this species occurs in Russian Karelia. Thus, *Aedes sticticus* is not included in the Finnish list.

***Culex apicalis* Adams, 1903**. Originally reported by Martini (1928) from Finland. All European reports of this Nearctic species before 1950 represent the closely related Holarctic species *Culex territans* (Becker et al. 2003).
